# Mistletoe Extracts during the Oncological Perioperative Period: A Systematic Review and Meta-Analysis of Human Randomized Controlled Trials

**DOI:** 10.3390/curroncol30090595

**Published:** 2023-09-06

**Authors:** Elise Cogo, Mohamed Elsayed, Sukriti Bhardwaj, Kieran Cooley, Christilynn Aycho, Vivian Liang, Peter Papadogianis, Athanasios Psihogios, Dugald Seely

**Affiliations:** 1 Patterson Institute for Integrative Oncology Research, Canadian College of Naturopathic Medicine, Toronto, ON M2K 1E2, Canada; elisec1234@gmail.com (E.C.); melsayed@thechi.ca (M.E.); sbhardwaj@ccnm.edu (S.B.); kcooley@ccnm.edu (K.C.); ca@nadarra.health (C.A.);; 2 Radiation Oncology Department, National Cancer Institute, Cairo University, Cairo 11796, Egypt; 3 The Centre for Health Innovation, Ottawa, ON K2P 0M7, Canada; 4 Pacific College of Health Sciences, San Diego, CA 92108, USA; 5 National Centre for Naturopathic Medicine, Southern Cross University, Lismore 2480, Australia; 6 School of Public Health, University of Technology Sydney, Ultimo 2007, Australia; 7 The Ottawa Hospital Research Institute, Ottawa, ON K1H 8L6, Canada

**Keywords:** anthroposophic medicine, cancer, European mistletoe, integrative oncology, mistletoe extracts, perioperative, naturopathic medicine, surgery, *Viscum album*

## Abstract

Background: We aim to evaluate the safety and efficacy of mistletoe extract (ME) use during the oncological perioperative period. Methods: Details registered a priori on PROSPERO (CRD42018086168). Results: Seven RCTs (comprising 663 participants in nine reports) and three nonrandomized studies were included. In five RCTs, ME was evaluated as adjunctive care and the control group had no additional intervention, whereas in two RCTs, ME was compared head-to-head against common cancer treatments (i.e., etoposide or bacillus Calmette-Guérin) with the intervention groups not receiving standard care. Meta-analyses found no evidence for a difference between ME and no added therapy for mortality and recurrence (RR, 95% CI: 1.00, 0.79–1.27; and 1.03, 0.79–1.33, respectively). Two RCTs reported beneficial effects of ME on immune cells, specifically natural killer cells, in colorectal cancer, and one RCT reported quality of life improvement. Two RCTs reported ME discontinuations due to adverse events and grade 3/4 toxicities. Nevertheless, no safety signals were detected from these 10 studies. Quality appraisal revealed a substantial risk of bias. Conclusions: Preliminary data are encouraging for mistletoe extracts, particularly in the context of colorectal cancer. However, the evidence is limited by the number of studies, an evaluation of different outcomes, and methodological limitations. Further high-quality research is warranted.

## 1. Introduction

Mistletoe is a parasitic plant that grows on trees without harming the host [1,2,3,4], and it has been widely used as part of traditional medicine in Europe and in other parts of the world to treat several health conditions [4,5,6]. In contemporary settings, the most common routes of administration are via subcutaneous (SC) or intravenous (IV) injection. Other routes of administration have also been reported [1,3].

The application of mistletoe was first proposed for use in cancer by Rudolf Steiner in 1920 [7]. Following this, the clinical use of mistletoe extract (ME) in cancer as an adjuvant tool has been further explored and expanded [8]. *Viscum album L.* extracts (VAEs, *V. album L.*; European mistletoe) are the most commonly used species due to their potential anti-cancer properties demonstrated in vitro [2,9].

There are many bioactive compounds in ME that have been characterized, including various lectins (I, II, III), viscotoxins, amino acids, flavonoids and polysaccharides [10]. Among these compounds, those believed to be most relevant to cancer-supportive care are mistletoe lectins and viscotoxins [11]. Viscotoxins are small proteins that mainly have cytotoxic (cell-killing) effects and possible immune system stimulatory properties [12], whereas Lectins are complex molecules consisting of both protein and carbohydrates and can change the biochemical characteristics of immune cells and modulate their functions by binding to their outside surface [13,14]. Mistletoe extract preparations have demonstrated both cytotoxic and immune-stimulatory effects against tumor cells in vitro [15,16,17,18].

The cytotoxic effect of ME and its constituents may occur by targeting two signaling pathways that regulate tumor proliferation resulting in apoptosis and cancer cell cycling, namely the PI3K/AKT (phosphatidylinositol 3-kinase/protein kinase B) and MAPK (mitogen-activated protein kinase) pathways [4,19,20,21,22]. Other mechanisms by which mistletoe may exert its cytotoxic effect are through the promotion of apoptosis [21], arresting cell cycling [22], inhibiting angiogenesis [23], generation of reactive oxygen intermediates (ROI) and inhibiting cellular protein synthesis [24,25,26].

Dendritic cells (DCs) and natural killer (NK) cells are the primary cell subtypes of the immune system that appear to be most positively affected by mistletoe. The use of *Viscum album* extracts (VAEs) on immature dendritic cells led to stimulation of both the maturation and activity of DCs in one study [27]. Similarly, Korean mistletoe lectins (*Viscum album coloratum*) have enhanced the maturation of bone-marrow-derived dendritic cells in vitro [28]. Tumor cells have been able to resist immune-mediated destruction by producing immunosuppressive substances that can reduce dendritic cell activity. In addition to enhancing dendritic cell maturation, mistletoe may counteract DC immunosuppression, as evidenced by a renal cancer cell model [29].

Another effect on the immune system demonstrated by MEs, particularly lectin-rich extracts, is its action on NK cells. This has been demonstrated through enhanced lysis of glioblastoma cells in an in vitro study [30], and in another study, an increase in natural killer cytotoxic activity among breast cancer patients after a single intravenous infusion of a *Viscum album* extract [31]. In cultures of human peripheral blood mononuclear cells exposed to ME the secretion of immune-system-enhancing cytokines, such as interleukin-1, interleukin-6, and tumor necrosis factor-alpha were increased [16,32].

Despite the benefits of surgery, it can have a profound impact on a person’s physiology and immune function and can negatively affect the course of cancer, including its recurrence. Surgery and cancer often lead to immunosuppression and, together, they can have an additive immunosuppressive effect and consequently lead to an increased risk of postoperative complications such as infections and potentially the spread of malignancy [15]. Immunomodulation (and the use of immunotherapy) in the perioperative period before, during or after surgery may have an important role in overcoming these risks [15].

The inflammatory response to surgery or trauma creates surgical inflammation [33,34,35]. The local inflammatory response in a surgical wound occurs in the early postoperative period and leads to the activation of the innate immune response. Consequently, there is an influx of neutrophils and monocytes into the wound and a major production of cytokines and chemokines [36]. In the postoperative period, a balance is required between pro-inflammatory cytokines and anti-inflammatory agents and cytokine release. This balance helps to restore and maintain hemostasis and prevent systemic inflammatory immune response (SIRS) or immunosuppression, respectively [36,37,38,39]. Mistletoe, through inhibition of the COX protein, may act as an anti-inflammatory agent in this setting [40].

Surgery could influence local recurrence and spread of cancer cells as shown from in vivo experimental research where the increased severity of surgical stress enhances the development and severity of the lung metastases [41]. Furthermore, animal studies suggest that stress hormones released in response to surgery could be a risk factor for cancer recurrence [42]. In addition, surgery promotes a hypercoagulable state [43,44]. This hypercoagulable state decreases activity of NK cells and increases the survival of tumor cells or decreases the clearance of the tumor cell, thus promoting metastases [44]. Moreover, low pre-operative NK cell activity was found to be associated with tumor recurrence in colorectal patients who underwent surgery [45].

Cell-mediated immunosuppression during the perioperative period could also help promote the development of metastases after surgery [46,47]. This suppression of cellular immunity following major surgery is transient, yet can be clinically significant [47]. In colorectal cancer patients who underwent surgery, NK cell activity dropped by day one postoperatively and only started to recover in the third week post-surgery; this correlated with the burden of the disease [45]. As a result, the early postoperative period is suggested to be an ideal time for immune-based interventions that reduce or protect against immunosuppression [46].

Of added interest in this area is that tumors produce anti-angiogenic as well as local pro-angiogenic activators [48]. Upon surgery, there may be a reduction in the production of more stable anti-angiogenic mediators like angiostatin or endostatin, as well as post-surgical production of angiogenesis signals, leading to potential angiogenesis of previously dormant micrometastases [49,50]. In this context, the use of anti-angiogenic therapies may in fact have a role to play in mitigating some of the risks of surgery [51].

Surveys suggest that mistletoe therapy remains a relatively popular choice for cancer patients [52,53]. However, despite increasing consumer familiarity and adoption of ME alongside other integrative medicine therapies in cancer care [54], ME has not been approved for use in cancer care in some jurisdictions [16]. Prior studies and related systematic reviews offer mixed or inconclusive conclusions regarding the effectiveness of ME, perhaps due to variations in preparations and their pharmacological properties. In addition, the inadequate methodological design of many studies creates more uncertainty as to the clinical utility of this therapy [55,56].

To address this gap, we aim to evaluate whether ME use in cancer patients around surgery may help overcome the undesired effects of surgery mentioned earlier and to identify any potential effects on the cancer itself. We have conducted this systematic review and meta-analysis to synthesize available evidence, determine the potential role of this therapy in the perioperative setting, and determine what additional research is needed. To our knowledge, this is the first systematic review and meta-analysis that evaluates the efficacy and safety of the use of ME in cancer patients during the perioperative period.

## 2. Materials and Methods

This systematic review is part of a larger umbrella project to prepare systematic reviews examining the use of natural health products among cancer patients during the perioperative period and was based on our institution’s initial prioritization exercise [57]. The current report describes the mistletoe section of this larger research agenda. The original protocol was registered *a priori* on the PROSPERO website (CRD42018086168) and has been described in two previous publications in detail [58,59]. The conduct of this systematic review followed the Cochrane Collaboration guidelines [60] and was written following PRISMA reporting guidance [61,62].

The inclusion criteria for this systematic review allowed for patients undergoing cancer-related surgery that evaluated mistletoe extracts, utilizing any method of administration, formulation, duration, and dose compared to placebo, standard care, or active control. The primary outcomes assessed were cancer recurrence, mortality, treatment response, remission, stable disease, and metastasis/disease progression. Secondary outcomes included postoperative complications, postoperative infections, adverse events, blood loss (perioperative blood transfusion), length of hospitalization, pain, fatigue, quality of life (performance status), anthropometric indices, immune cell measurements, inflammatory markers levels, and cancer biomarkers. We included published and unpublished reports concerning the use of mistletoe in cancer patients during the perioperative period.

To assess for efficacy outcomes, only randomized controlled trials (RCTs) were included, whereas for safety outcome evaluation, non-randomized controlled clinical trials and controlled observational studies, in addition to the RCTs, were included for broader data capture. Non-English reports and studies examining the use of concomitant conventional treatment (i.e., chemotherapy, radiotherapy, etc.) with mistletoe were excluded.

MEDLINE, Embase, and Cochrane CENTRAL databases were searched for human studies in English from inception to 14 January 2023. The Peer Review of Electronic Search Strategies (PRESS) checklist [63] was used by an expert librarian (JM) to peer review the literature search (the Supplementary File includes the MEDLINE search strategy). Furthermore, searches were performed within the clinicaltrials.gov (accessed on 17 July 2023) trials registry, the Natural Medicine (formerly Natural Standard) database, and Health Canada’s Natural Health Product Monographs as a supplemental grey literature search. To identify any other potentially relevant studies, we scanned the list of references of the included studies and the related systematic reviews.

Screening studies for eligibility, data extraction, and risk of bias appraisal of the RCTs and quality assessment of the non-RCT and observational studies were conducted independently by two research team members with a consensus approach taken to resolve any discrepancies. The original (2011) Cochrane Risk of Bias tool was used for appraisal of the RCTs [64]. For the assessment of quality for non-RCT and observational studies, the Newcastle–Ottawa Scale was applied (NOS) [65].

Data extraction was performed in Excel sheets; descriptive synthesis of the result was conducted first and then evidence summary tables were created. Meta-analyses were conducted when possible and appropriate using STATA 12 [66]. The guidelines from the Council for International Organizations of Medical Sciences (CIOMS Working Group X) and Cochrane were followed in dealing with statistical heterogeneity and in conducting the meta-analysis [60,67]. Forest plots were created to graphically present the main results from the meta-analysis. The relative risk (RR) for binary outcomes and mean difference (MD) for continuous outcomes were calculated for each trial, when possible, to represent the summary effect measures.

## 3. Results

### 3.1. Included Studies

**Figure 1:**
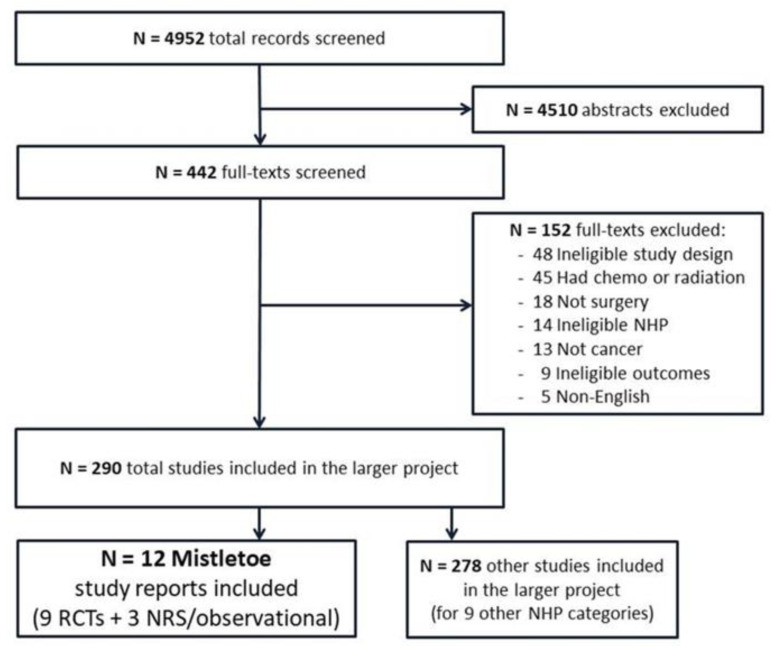
Study flow diagram.

The details of the literature search, including title and abstract screening, full-article screening, reasons for exclusion within the larger project, and the number and type of studies for the mistletoe analysis, are presented in the study flow diagram in Figure 1. In summary, the searches retrieved 4952 records (abstracts) for the larger project, and 442 full-text articles were screened. Overall, 290 studies were eligible and included. For the mistletoe section, twelve relevant articles were included reporting on ten studies comprising seven RCTs in nine reports [15,68,69,70,71,72,73,74,75] and three observational cohort studies [76,77,78]. The seven RCTs included 633 participants in total.

The trial and patient characteristics of the included RCTs and the non-RCTs are reported in Table 1 and Table S1, respectively. Additionally, details of the intervention and control arms of the RCTs and non-RCTs are outlined, respectively, in Table 2 and Table S2. Summary characteristics across all studies are presented in Table S3. The RCTs’ publication dates ranged from 2001 to 2020, with all seven RCTs initially published between 2001 and 2009. Two additional updates from one RCT [71] were published in 2014 and 2020 [72,73]. Sample sizes of the RCTs ranged between 20 and 204 participants. Three RCTs (43%) had a sample size of less than 50 patients. Six RCTs were conducted in Europe, and one was conducted in Egypt. Funding was reported in six RCTs; three (43%) were publicly funded, one RCT (14%) was privately funded, one RCT (14%) had a mix of public and private funding, and one RCT (14%) did not have a funding source. In one RCT (14%), funding was not reported.

The cancer populations studied in the seven RCTs included two (29%) for bladder [68,69], two for digestive tract/colorectal cancer [15,74] and one each (14%) for osteosarcoma [73], melanoma [70] and head and neck cancer [75]. The mean or the median age of the study populations among six RCTs ranged between 33.9 and 71 years old. One RCT did not report the mean or median age of the sample’s population for each arm, but rather reported that the mean age in the ME group was 62 years (range 37–87 years) with no differences between the groups regarding the age. The proportion of females among six RCTs ranged between 9% and 45%, and no data regarding the percentage of the female population were reported in one RCT.

ME was administered during the postoperative period in four RCTs (57%), during both pre-and postoperative periods in two RCTs (29%), and intra-operatively in one RCT (14%). Duration of administration in the RCTs ranged from a single intravenous application intra-operatively (administered over a one-hour time period) [74] up to 60 weeks (15 months, given in cycles with 12 weeks of treatment followed by 4 weeks without treatment) [75].

The majority of RCTs (57%) compared mistletoe to usual care (i.e., with no added treatment) [15,68,70,74,75]. However, in two RCTs (29%), mistletoe was not evaluated as adjunctive but instead compared against a common cancer treatment (i.e., etoposide or bacillus Calmette-Guérin [BCG]), so the intervention group did not also receive standard care [69,73]. The subcutaneous (SC) administration of mistletoe was the most common route of administration with five RCTs (72%) utilizing this application method. One RCT (14%) used mistletoe intravenously (IV) intra-operatively, and one RCT reported intravesical application in bladder cancer patients.

### 3.2. Methodological Quality Assessment and Risk of Bias

**Figure 2:**
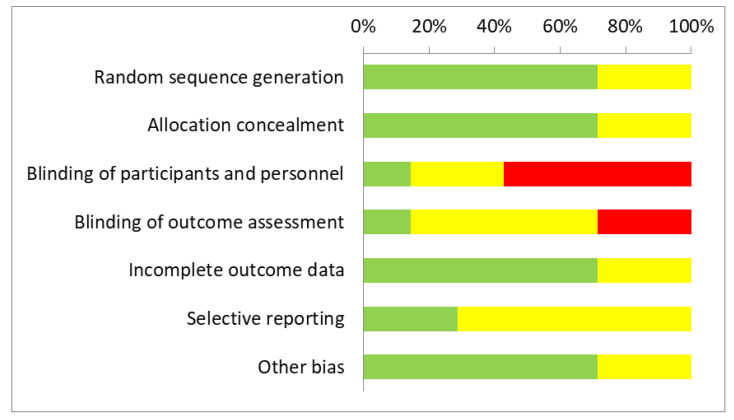
Aggregate risk of bias across studies. Green = low risk; yellow = unclear risk; red = high risk of bias.

Table 3 and Figure 2 depict the risk of bias assessments for each RCT and the aggregate summary across the RCTs, respectively, using the Cochrane Risk of Bias tool. A substantial risk of bias was detected among all the included studies using the methodological quality appraisals as none of the RCTS were judged to have a low risk of bias overall. Four (57%) RCTs were rated as high risk of bias for at least one domain among the seven assessment elements. The other three (43%) RCTs had an unclear risk of bias for at least one domain. The elements that scored worst were related to blinding as none of the RCTs reported being double-blinded, suggesting potential performance and detection bias. In addition, the majority of the studies were unclear for selective reporting as trial registration (or an accessible protocol) was not cited, indicating possible reporting bias.

Table 4 presents the quality assessment of the three non-RCTs using the Newcastle–Ottawa Scale (NOS). Methodological quality varied considerably, with the Augustin study [77] scoring best with nine out of nine total stars. However, the “Outcome” domain demonstrated several limitations in the Bussing and Elsasser-Beile studies [76,78], in particular due to short follow-up times. Moreover, the “comparability of cohorts on the basis of the design or analysis” item was inadequate as these two studies did not control for important potential confounding. Regarding publication bias, while there were too few studies to reliably create a funnel plot, this concept is incorporated in the original Cochrane ROB tool (i.e., non-reporting, missing results).

### 3.3. Results of the RCTs by Comparators

The outcomes of the RCTs are presented according to the control group and divided into these three relevant comparison categories:

1. Mistletoe versus no added treatments;
2. Mistletoe versus etoposide;
3. Mistletoe versus BCG.

#### 3.3.1. Mistletoe Versus No Added Treatments (5 RCTs)

Mistletoe was compared to no added treatment in five out of seven RCTs (72%) [15,68,70,74,75].

##### Primary Outcomes (First Comparison)

Mortality, Recurrence and Metastasis

**Figure 3:**
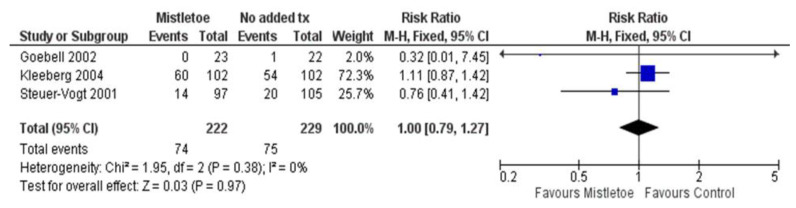
Forest plot of RCTs meta-analysis on mortality, Goebell 2002 [68], Kleeberg 2004 [70], Steuer-Vogt 2001 [75].

Three RCTs in this comparison evaluated mortality and were included in the meta-analysis [68,70,75]. Supplementary Table S4 presents the mortality outcome data and Figure 3 displays the forest plot of the meta-analysis for mortality. A fixed-effects model was applied as there was low statistical heterogeneity between studies (I^2^ = 0%). The follow-up duration among the studies ranged between 18 months and 8 years, with one study noting a median follow-up period of 4 years [75]. Meta-analysis found no evidence of a difference in survival between ME and no added treatment (number of studies, k = 3; total sample size, N = 451; RR = 1.00; 95% CI, 0.79 to 1.27; *p* = 0.97). In addition, over the Kleeberg 2004 RCT [70] 8-year follow-up period in melanoma patients, there were slightly more deaths in the ME group (59% vs. 53% of participants), but the difference was not statistically significant, and the total number of deaths among the three RCTs was very similar (74 vs. 75, respectively).

**Figure 4:**
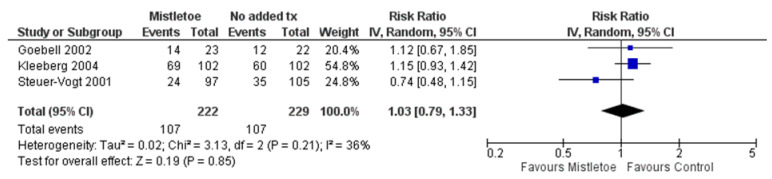
Forest plot of RCTs meta-analysis on recurrence, Goebell 2002 [68], Kleeberg 2004 [70], Steuer-Vogt 2001 [75].

Figure 4 presents the forest plot of the meta-analysis for cancer recurrence. Similar to the mortality outcome for this comparison, three studies reported on cancer recurrence (the same studies reported on mortality outcome) and were included in the meta-analysis [68,70,75]. Follow-up time for assessing cancer recurrence varied between 18 months to 8 years. A random effects model was applied for meta-analysis as there was moderate statistical heterogeneity between the studies (I^2^ = 36%). The comparison of mistletoe versus no added treatment yielded no evidence of a difference in cancer recurrence (k = 3; N = 451; RR = 1.03; 95% CI, 0.79 to 1.33; *p* = 0.85). Metastasis outcome data were included in the recurrence meta-analysis data mentioned above, which revealed no evidence of a difference between the mistletoe and control group for recurrence (including metastasis). Two RCTs in this comparison reported on the incidence of distant metastasis. No other extractable data were reported within the five RCTs for this comparison regarding the other primary outcomes of the systematic review including treatment response, remission, and stable disease.

##### Secondary Outcomes (First Comparison)

Quality of Life

One RCT reported on quality of life for this comparison at 60 days follow-up. Enesel 2005 found that ME (Isorel) administration (n = 40) improved self-reported functional impairment score (measured with the Karnofsky Performance Index (KPI)) compared to baseline value by 22% (*p* < 0.01) in participants with digestive tract cancers (51% colorectal), while the KPI decreased by 13% (*p* < 0.05) in the control group (n = 30) [15]. Quality of life results, including for fatigue and pain subscales, reported for all RCTs are presented in Table S4.

Immune Cells and Inflammatory Markers

Two RCTs investigated the effects of ME on immune cells and inflammatory markers for this comparison. The results of the immune cells and inflammatory markers for all RCTs are presented in Table S4. Enesel 2005 evaluated over 15 blood parameters in digestive tract cancer participants at 28-day follow-up (i.e., 14 days postoperative). Compared to baseline values (i.e., those reported as within-group relative percent change), the ME arm had increased numbers of NK cells (by 44%, determination method using CD2-CD3) and T and B cells. In particular, the increases were seen in T-helper (CD4) cell (by 18%), complement (by 20–30%), IgA (by 27%), and IgM (by 23%) values after Isorel injections for 2 weeks pre-operatively and 2 weeks postoperatively. In contrast, the no added treatment control group had a non-significant decrease in NK values and had a greater increase in total peripheral leukocyte (WBC) count compared to their baseline mean values. Notably, CD8 (T-suppressor) values in the ME group showed a significant decrease (by −7%) compared to baseline, whereas in the control arm, there was a slight but non-significant increase (by 3%) [15]. Schink (2007) examined intra-operative ME use in colorectal cancer and found a smaller drop from baseline in NK cell activity on day 7 postoperatively in the mistletoe group versus the control (*p* = 0.04). In regard to monocyte expression of HLA-DR antigen in this RCT, there was no difference seen between the mistletoe and control groups (*p* = 0.46) [74].

Blood Loss

One study reported on perioperative blood transfusion (erythrocyte administration) in this comparison (Table S4) and found no significant difference in the number of perioperative blood transfusion events: 1 in 12 patients in the ME (Iscador® M) group versus 3 in 14 patients in the no added treatment group (*p* > 0.05) [74].

Other Complications and Adverse Events

For this comparison, four RCTs [68,70,74,75] reported on adverse events (AEs) and other complications while one RCT did not report on adverse events [15]. Table 5 presents detailed adverse event data reported across all RCTs. Treatment discontinuations due to grade 3 or 4 (WHO) toxicities in one RCT among melanoma patients were more frequent in the ME arm versus no added treatment (5 out of 102 vs. 0 out of 102 participants, respectively) [70]. One RCT reported no adverse events after application of mistletoe [68]. Another RCT found similar numbers between groups for AEs during surgery, serious AEs, and postoperative AEs [74]. The final RCT did not report any AEs in the control arm, whereas in the ME group, 16 out of 97 head and neck cancer participants refused further injections because of mistletoe-induced AEs. Local and/or systemic reactions around the time of injection were reported in 47 out of 97 (with rubor and prurigo being the most common) at the start of therapy and at 32 weeks or later in 4/97 patients. Among the same ME cohort, general drug reactions occurred in 4 out of 97 patients (e.g., melalgia, fever ≤ 39 °C, sleeplessness, tiredness, coldness or heat sensation, sneezing) [75]. No extractable data were reported in these five RCTs regarding the other secondary outcomes of the review, namely postoperative infection, hospitalization length of stay, anthropometric data (BMI and body weight), cancer biomarkers, fatigue, and pain.

#### 3.3.2. Mistletoe versus Etoposide (Second Comparison (1 RCT)

##### Primary Outcomes (Second Comparison)

The Longhi et al. RCT, which is reported across three publications, includes a report on a 12-month trial follow-up period among a small sample of patients with osteosarcoma randomized to either ME (no other conventional treatment given) or etoposide after surgery for a second cancer relapse in the lung. The number of deaths due to any cause was 0 out of 9 patients in the mistletoe group versus 3 out of the 11 patients (27%) in the etoposide group (*p* > 0.05). In addition, the number of patients with a relapse after one year was 4 out of 9 (44%) in the mistletoe group versus 8 patients out of 11 (73%) in the etoposide group (*p* > 0.05) [71,72,73].

#### Secondary Outcomes (Second Comparison)

Quality of Life

Quality of life was assessed in the Longhi (2014) RCT using the EORTCBB QLQ-C30 questionnaire in adults or the Pediatric Quality of Life Cancer Module Acute version 3.0 (PedsQL) in patients less than 18 years old. There was a trend for positive effects from using ME on most of the parameters and in particular for the global QoL scale, functional scales including the physical and social functioning, and financial functioning, in addition to the symptom items of fatigue, pain, and dyspnea. In contrast, with the use of etoposide, improvement was only seen for social functioning and there were deteriorations in the pain and nausea/vomiting symptom items (Table S4) [72].

Immune Cells and Inflammatory Markers

Longhi (2020) reported in a small RCT that administered ME for 12 months versus etoposide for 6 months and compared lymphocyte subpopulation counts for CD3, CD4, and CD56 (NK) cells compared to baseline. In the ME arm, these all increased at 6 and 9 months and then decreased at 12-month follow-up (e.g., for CD56, mean and SD: –24.59 ± 180.06 mm^3^). Notably, only by the last timepoint had these increased in the standard drug group (N = 9; Table S4) [73].

Other Complications and Adverse Events (RCTs)

The etoposide drug group experienced more total adverse events and treatment discontinuations than the mistletoe extract arm in this RCT, but serious AEs (i.e., hospitalizations due to surgery) were similar. Details of the AEs and other complications are presented in Table 5. The following secondary outcomes of the review—postoperative infection, hospitalization length of stay, anthropometric data (BMI and body weight), blood loss, and cancer biomarkers—were not reported in this RCT.

#### 3.3.3. Mistletoe versus BCG (1 RCT) (Third Comparison)

##### Primary Outcomes (Third Comparison)

Recurrences and Metastasis

Ibrahiem (2009) only reported results in a conference abstract format and reported recurrence rates after using intravesical mistletoe versus BCG (route not reported) in superficial bladder cancer patients (Ta, T1 of Grade 1–2) following TURBT (transurethral resection of bladder tumor). Five patients in each group developed invasive bladder cancer (*p* > 0.05). The number of local recurrences was 9 out of 30 patients in the BCG group versus 22 out of 30 patients in the mistletoe group (*p* = 0.003). Stage and grade progression occurred in 14 patients (47%) in the mistletoe group versus 4 patients (13%) in the BCG group (*p* = 0.01). One patient in the mistletoe group developed lymphatic and hepatic metastases versus no patients in the BCG group (*p* > 0.05) [69]. This RCT did not report on the rest of the systematic review’s primary outcomes.

##### Secondary Outcomes (Third Comparison)

Other Complications and Adverse Events (third comparison)

Self-limited local skin reaction after first SC dose was reported in all patients with ME, and the authors only reported that the intravesical ME was “tolerable and safe” without providing further details. A painful bladder sensation was reported in 23 of the 30 patients in the BCG group. Further details of the AEs and other complications are presented in Table 5. This RCT did not report on the other secondary review outcomes of interest.

### 3.4. Results of Non-Randomized and Observational Studies

Three non-RCTs were additionally included for further safety evaluation, comprising two non-randomized clinical trials and one observational controlled cohort study in participants with malignant melanoma (N = 686), breast cancer (N = 101), and superficial bladder cancer (N = 48) (Tables S1–S3). The primary outcomes of mortality, recurrence, and metastasis were only reported in one study each, and no safety concerns were seen (data presented in Supplementary Table S5). The only secondary review outcome of interest reported across these three studies, other than adverse events (AEs), was immune and inflammatory markers in one study, which found that C-reactive protein, leukocytes, lymphocytes and the main subsets (i.e., T cells, B cells, natural killer cells, CD4+, CD8+ CD28 and CD8+ CD28+ CD25+ T cells, and the cytokines interleukin 6) were similar between the ME and no added treatments groups [78]. Table S5 presents the AE data reported in these studies. Two non-randomized clinical trials reported that there were no mistletoe-related side effects among 80 total participants in the ME groups [76,78]. Augustin (2005) conducted a controlled cohort study evaluating 329 participants who had received mistletoe extract for a median of 30 months and reported 11 (3%) systemic adverse drug reactions (ADRs) attributed to the mistletoe treatment, 42 local injection site reactions, and 6 mistletoe treatment terminations due to ADRs [77].

## 4. Discussion

This comprehensive systematic review included 12 publications, in which seven RCTs with 663 participants were synthesized to evaluate ME efficacy in the context of perioperative cancer care. The results of this systematic review with meta-analysis suggest a possible benefit of ME use for patients with cancer undergoing surgery, with a positive impact on immune function (NK cells) and also on quality of life (functional impairment). This is primarily from data from two RCTs comparing ME against no added therapy. The evidence, however, is fairly sparse, and the risk of bias is unclear or high, mainly due to a lack of blinding and/or placebo/sham controls. Methodological limitations weaken the strength of the findings and further investigation is warranted using study designs that address these limitations.

The seven included RCTs were published between 2001 and 2009, and two additional updates of an original RCT were published in 2014 and 2020. Additionally, three non-randomized controlled studies were included on the safety of using ME in this context. There was clinical and methodological heterogeneity across the RCTs. The cancer types studied in the RCTs included the following: bladder (two), digestive tract/colorectal (two), osteosarcoma (one), melanoma (one), and head and neck cancer (one). Various extracts of mistletoe were used and two RCTs did not report the specific extract administered. The synthesis of outcomes of this SR was based on three comparisons relating to the control arm of the RCTs. Five RCTs (72%) compared ME to no additional treatment, and three reported on mortality and recurrence and were able to be included in the meta-analyses of mortality and recurrence. In two RCTs (29%), ME was not used as an adjunctive element to therapy but instead was compared against a common cancer treatment (i.e., etoposide or bacillus Calmette-Guérin (BCG)), wherein the intervention group did not receive standard care. Five RCTs (72%) administered ME subcutaneously, and four out of the seven RCTs (57%) used it during the postoperative period. Duration of therapy in the RCTs ranged from intra-operatively only up to over a 60-week period (cycling with breaks). All these variations contribute to the limitation of the results of the current SR.

From the current SR, the effect of ME on mortality and recurrence, including metastasis, was inconsistent, and the evidence of these outcomes needs to be explored in future high-quality research studies. While meta-analysis for mortality and recurrence revealed no significant difference between MEs and no added treatment [68,70,75], one small RCT in osteosarcoma patients, Longhi et al., 2009, reported favoring but not significant relapse and survival results at 12 months using ME versus etoposide [71]. The increased relapse rate and disease progression in an RCT that administered intravesical ME in superficial bladder cancer patients versus BCG was attributed to the relatively insufficient dose of ME used [69]. In contrast, there were some positive effects on quality of life and immune and inflammatory markers from the application of ME in three comparisons (no added treatment, colorectal patients, and active treatments) [15,71,73,74].

The details of adverse events were poorly reported across the RCTs. Overall, there were no significant concerning side effects, serious or severe adverse events, and/or postoperative complications from using MEs in cancer patients perioperatively [68,69,74]. One study even reported a better overall side effect profile with the use of MEs [71]. However, it is important to note that two RCTs highlighted concerns that were related to the application of MEs compared to no added interventions [70,75]. Kleeberg et al., 2004, reported that among patients with melanoma, treatment discontinuation due to grade 3–4 toxicities was reported in 5 out of 102 (5%) patients who received Iscador® M in comparison to 0 out of 102 (0%) patients in the no added treatment group [70]. Similarly, Steuer-Vogt et al., 2001, in addition to the reported local and systemic side effects resulting from mistletoe SC injections into the abdominal wall, reported 16 patients who received Eurixor® (mistletoe extract) who refused further injections because of mistletoe-related AEs (without a precise definition of these AEs) [75]. One controlled cohort study conducted by Augustin in 2005 evaluating 329 participants who had received mistletoe extract for a median of 30 months reported 11 (3%) systemic adverse drug reactions (ADRs) attributed to the mistletoe treatment, 42 local injection site reactions, and 6 mistletoe treatment terminations due to ADRs [77]. No safety concerns overall were identified from the non-randomized and observational trials [76,77,78].

There is a paucity of data from other systematic reviews specifically reporting on the efficacy and safety of using MEs in oncology patients during the perioperative period; moreover, the use of MEs among oncology patients in general is still an area of controversy. There are many reports on MEs used alongside conventional and standard treatment in oncology patients. Most of these RCTs evaluated quality of life, symptom management, and side effects from standard treatment and reported positive effects from the use of MEs for these outcomes [15,72,79,80,81,82,83,84,85,86,87]. These results are also supported by a recent SR [88]. Survival results across studies were mixed; some reported benefits [72,73], and some did not [84,89]. The SRs of the clinical trials reported methodological flaws regarding the included RCTs, making the interpretation of results challenging [2,90,91,92,93,94]. In addition, there is some controversy about the results of some of these SRs [92,93,95,96,97]. In general, the methodological quality appraisals of our SR revealed a substantial risk of bias across all included RCTs with none judged to have a low risk of bias overall (Figure 2, Table 3 and Table 4). The high risk of bias is consistent with reports from other SRs [88,93,94]. Methodological quality varied considerably between the non-randomized studies in the current SR, with the Augustin study [77] scoring best with a quality score of 9 out of 9 according to the NOS. However, the “Outcome” and “comparability of cohorts on the basis of the design or analysis” domains demonstrated several limitations and inadequacy, respectively, in the Bussing and Elsasser-Beile studies [76,78].

The strengths of the current SR and MA include being the first SR to address a particular oncology population (perioperative oncology patients) and examine the use of MEs among them, comprehensiveness of the review process, providing an up-to-date review of the literature, a priori protocol registration, and transparency in reporting the outcomes. Furthermore, all the steps of this SR were duplicated to ensure accuracy. In addition, the inclusion of the non-randomized trials for safety data and information regarding the application of MEs in cancer patients during the perioperative period strengthened the review [98,99]. The main limitation of the methodology was in excluding non-English reports and not searching Asian databases, which introduced a language bias.

The perioperative period in cancer patients is challenging as it usually includes major surgeries, stressful and lengthy preparations, hospital admission, and prolonged recovery [98]. Surgically induced stress, the resultant inflammatory response, and a weakened immune response might promote angiogenesis, tumor shedding and metastasis, and proliferation of tumor growth factors [100]. Aiming for a favorable immune balance during the perioperative period is essential for long-term benefits, including the survival of cancer patients [101]. One strategy to overcome these challenges and achieve the immune balance is combining cancer surgery and immunotherapy [100]. However, as a complex treatment strategy, patients may develop more pronounced side effects and limitations before having any benefit. Furthermore, immunomodulation may have adverse effects on wound healing, recovery, and risk of infection, and the benefits, especially among cancer patients, need to be weighed against the risks [100].

Despite the limitations of the evidence we have reviewed, there is a signal of positive effects from using MEs during the perioperative period in cancer patients, in particular for immune support and quality of life. There is sufficient evidence to call for more research based on the recent guidelines in this area, in particular for colorectal cancer during the perioperative period. The low risk of side effects seen from the available research and the potential for reduced immunosuppression, in addition to MEs’ cytotoxic effects, provide a compelling basis for further evaluation [4,19,21,22,23,24,25,26].

## 5. Conclusions

Our systematic review suggests a possible benefit of using ME with respect to positive impacts on the immune system (especially NK cells), and also potentially on quality of life, in patients with cancer during the perioperative period. With respect to survival, results showed no evidence of a difference compared to no added therapy and were inconsistent. Nevertheless, the evidence base is fairly sparse due to different outcomes evaluated across studies, and has methodological limitations rated as either unclear or high risk of bias. Further high-quality research is warranted to investigate the use of ME in the perioperative setting, especially in patients with colorectal cancer.

## Figures and Tables

**Table 1:** Characteristics of included RCTs.

Study Author, Year (Study Period)	Country	Sample Size ^a^	Funding	Surgical Period of Mistletoe Exposure ^b^	Post-op Mistletoe Initiation Timing ^b^	Cancer Types	Cancer Severity	Age (y, var.)	% Female	Adjunct tx (%)
Schink, 2007 (2002–2004) [74]	Germany	32	public	Intra-operative	NA	Colorectal cancer	primary and locally relapsed, stages II–IV	71 (NR)	45	Antibiotic (NR)
Enesel, 2005 (NR) [15]	Romania	70	NR	Mixed periods	NA	Digestive tract cancers (51% colorectal)	NR	NR (NR)	43	NR
Kleeberg, 2004 (1988–2003) [70] ^c^	13 countries: Germany; France; Switzerland; Austria; Belgium; Great Britain; Yugoslavia; Israel; Czechia; Estonia; Spain; Greece; Poland	204	mixed public and private	Post-operative	within 6 weeks	melanoma	High-risk stage IIb (thickness > 3 mm) and stage III (positive lymph nodes) without distant metastasis (49% were Stage Iib)	52 (range 14–84)	41	NR
Goebell, 2002 (NR) [68]	Germany	45	public	Post-operative	2 weeks postop	bladder cancer	pTa G1–2	65 ^d^ (NR)	27	NR
Ibrahiem, 2009 (2006–2008) [69] ^e^	Egypt	60	none	Post-operative	1–2 weeks postop	Superficial bladder cancer	Ta or T1; 82% were Grade 2	56.1 ^d^ (NR)	NR	NR
Steuer-Vogt, 2001 (1993–2000) [75] ^f^	Germany	202	public	Mixed periods ^f^	NA	Head and neck cancers	All stages	58 (range 30–70)	9	Antibiotic (88%)
Longhi, 2020 ^g^ (2007–2019) [73] ^g^	Italy	20	private	Post-operative	mean 1.9 (range 0.7–5.9) months	Osteosarcoma	2nd relapse in the lung (stages I–III)	33.9 (range 11–65)	45	NR

^a^ Number of patients randomized. ^b^ Indicate when mistletoe was started after surgery. ^c^ The study design included 2 trials together with 4 total arms but reported mistletoe results versus the control group only (not including the recombinant interferon-alpha and -gamma (rIFN-α2b and IFN-γ) arms. Study age is a median and pertains to all study arms combined. Not all patients followed up for 11 years (due to trial ending and lost to follow-up), but the median follow-up of 8.2 years was for all arms combined in the 2 trials (NR for just the mistletoe trial). ^d^ Age reported by median. ^e^ Conference abstract. ^f^ Mistletoe was started before surgery (1 ± 4 days before surgery), which was achieved in 74% of patients. Stratum A: Surgery-only patients (Data was only extracted from Stratum A). Stratum B: Higher-stage head and neck cancer patients who received surgery and postoperative radiotherapy. ^g^ Study closed early in 2011 due to insufficient recruitment. Only the RCT portion of the trial over the 1st 12 months was extracted from all reports (the longer follow-up is more like an observational study due to multiple protocol changes). This also includes the other reports Longhi 2014 and Longhi 2009. Abbreviations: NA = not applicable, NR = not reported, tx = treatment, var = variance, y = year.

**Table 2:** Interventions and comparators evaluated in the RCTs.

Study Author, Year	Group/ Brand Name	Characteristics of the Intervention and Control	Dosage Frequency	Route of Admin	Tx Duration Pre-op	Tx Duration Post-op	Tx Duration TOTAL
Schink 2007 [74]	Iscador® M special (Weleda, Schwäbisch Gmünd, Germany)	Mistletoe extract, 50 mg, IO	once	IV	NA	NA	Over 1 h
	Control	no added tx					
Enesel 2005 [15]	Isorel® A ^a^ (*V. album* grown on abietis extract)	Mistletoe extract, 2 vials (60 mg/mL, but the volume per vial was not reported). Protocol: in the 1st pre-operative week, 1 vial, then 2 vials, then 3 vials every second day and in the 2nd pre-operative week 3, then 2, then 1 vial; and postoperative treatments were repeated in the same manner.	3 × per week	SC	2	2	4 weeks
	Control	no added tx					
Kleeberg 2004 [70]	Iscador® M (*Viscum*, *mali* extract)	Mistletoe extract, the dose and the unit were not mentioned. Protocol was started at dose 0 and then was escalated every other day over 2 weeks starting from 0.01 to reach to 1.0 mg/mL, followed by a 3 day break. Treatment then resumed with 14 doses of 20 mg/mL over 28 days, followed by 7 days of no treatment. Volume per dose NR, but presumption is that 1 mL was given per dose.	3 × per week	Injection	NA	12	12 months or until tumour progression
	Control	no added tx					
Goebell 2002 [68]	*V. album lectin* extract (brand name NR)	Standardized to mistletoe lectin I (galactoside-specific lectin), 1 mL	2 × week	SC	NA	9 months, 2 cycles of 3 months of treatment separated by a 3-month break	9 months
	Control	no added tx					
Steuer-Vogt 2001 [75]	Eurixor® (biosyn Arzneimittel GmbH, Fellbach, Germany) (mistletoe extract)	Mistletoe lectin-1 (ML-1), 1 ng of ML-1 per kg body weight	2 × week	SC	1–4 days	60 weeks	60 weeks ^b^
	Control	no added tx					
Ibrahiem 2009 [69]	Mistletoe extract (brand name was NR)	Lectin concentration, 10.000 ng ^c^	NR	Intravesical	NA	NR	NR
	Control	Bacillus Calmette–Guérin (BCG), standard therapy ^c^	NR	Intravesical ^d^	NA	NR	NR
Longhi 2020 [73], Longhi 2014 [72], Longhi 2009 [71] ^f^	Iscador® P (*V. album fermentatum* Pini extract)	Mistletoe, 20 mg ^e^	3 × per week	SC	NA	12 months	12 months
	Control	Etoposide, 50 mg/m^2^	QD	oral	NA	6 months; 3 weeks per cycle, 6 cycles, separated by 1-week break	6 months

^a^ abietis—firtree; *Viscum album* extract by Novipharm GmbH, Pörtschach, Austria” was applied in this trial. The fresh mistletoe plant grown on firtree was used to prepare the extract by cold water extraction (60 mg of the whole plant for 1 mL of water). No homogenization or fermentation was applied. Extracted dose is an average. ^b^ Mistletoe extract was given in cycles, each consisting of 12 weeks, followed by a break of 4 weeks between 1 and 2, 2 and 3, and 3 and 4 cycles. ^c^ Course and dose were given according to the prescribed regimen (prescribed regimen was not mentioned in the abstract). ^d^ Standard treatment, given according to prescribed regimen, details of the injection were not reported for the BCG. ^e^ The protocol consisted of a starting dose; 2 boxes of series 0 (14 vials, seven vials in each box; 2 vials for each of 0.01 mg and 0.1 mg doses, and three vials of 1 mg), then two boxes of series I; 14 vials (2 vials of each of 0.1 mg, and 1 mg doses, and three vials of 10 mg dose per each box). Further treatment with series II (1, 10, and 20 mg) was given until the 12th month. The site of injection was the abdomen. ^f^ All these three citations were on the same patient cohort undergoing the study but with different outcomes. Abbreviations: NA = not applicable, NR = not reported, post-op = postoperatively, Pre-op = pre-operatively, IO = intra-operative, IV = intravenous, SC = subcutaneous, tx = treatment.

**Table 3:** Cochrane risk of bias appraisal of each RCT ^a^.

Author Year	Random Sequence Generation (Selection Bias)	Allocation Concealment (Selection Bias)	Blinding of Participants and Personnel (Performance Bias)	Blinding of Outcome Assessment (Detection Bias)	Incomplete Outcome Data (Attrition Bias)	Selective Reporting (Reporting Bias)	Other Bias
Enesel 2005 [15]	?	?	-	-	?	?	+
Goebell 2012 [68]	+	+	+	+	+	?	+
Ibrahiem 2009 [69]	?	?	?	?	+	?	?
Kleeberg 2004 [70]	+	+	?	?	?	+	+
Longhi 2020 [73]	+	+	-	-	+	+	?
Schink 2007 [74]	+	+	-	?	+	?	+
Steuer-Vogt 2001 [75]	+	+	-	?	+	?	+

^a^ Green “ + ” = low risk; yellow “?” = unclear risk; red “-” = high risk of bias.

**Table 4:** NOS quality assessment of non-RCTs ^a^.

Study Author, Year	Selection	Comparability	Outcome
Augustin, 2005 [77]	★★★★	★★	★★★
Bussing, 2005 [78]	★★★	★	★★
Elsasser-Beile, 2005 [76]	★★★	★	★

^a^ Based on the Newcastle–Ottawa Scale scoring guide, the stars indicate that one of the required quality elements was judged as being present, so a higher star rating (out of 9 maximum) represents higher methodological quality.

**Table 5:** Other complications (OC) and adverse events in RCTs.

Study Author, Year	Tx Name	Sample Size	Follow -Up Time and Unit	All Adverse Effects (AEs)/Most Common AEs (N) (Definition)	Serious AEs (N) (Definition	Severe AEs (N) (Definition)	Surgical Complications (N) (Definition)	Drug Reduction /Treatment Discontinuation Number (Definition)	OC (N) (Definition)
Longhi, 2014 [72]	Mistletoe	9	12 mon.	(16) (definition NR)	(2) (post -operative hospitalization)	(5) (definition NR)	NR (NA)	(0) Dose reduced (definition NR) (2) Medication discontinued (definition NR) (1) Medication continued after interruption (definition NR)	(2) Adverse drug reactions (Local erythema and hypotension (1))
	Etoposide	10	12 mon.	(69) (definition NR)	(3) (hospitalizations due to surgery (1) and pneumonia ^a^ (2)). 3	(26) (definition NR)	NR (NA)	(5) Dose reduced (definition NR) (4) Medication discontinued (definition NR) (18) Medication continued after interruption (definition NR)	(47) Adverse drug reactions (Including G2, G3 hematologic toxicity, G-CSF was necessary in 3 patients, 1 needed blood transfusion for G4 anemia. Most frequent ADRs were neutropenia, anemia, leukopenia, nausea, alopecia)
Schink, 2007 [74]	Iscador® M special (mistletoe extract)	11	7 days	NR	(0) (definition NR )	NR	(4) (AEs during surgery (hypotension (2), hypertension, circulatory instability) (5) (post-op AEs UTI (2), erythocytouria, body temp >38 °C and C-reactive protein >10 mg/dL, Tension bulla beneath bandage of peridural catheter)		(0) Allergic predisposition to mistletoe extract (hypersensitivity reaction to test injection dose pre-op of mistletoe (0.1 mg)
	No added tx	11	7 days	NR	(1) (anastomosis leakage requiring 2nd surgery)	NR	(4) (AEs during surgery hypotension) (4) (post-op. AEs (hypertension (2), erythocytouria, suppuration of surgical wound)	NR	NR
Enesel, 2005 [15]	No added tx	30	NR	NR	NR	NR	NR	NR	NR
	Isorel® A (*V. album* grown on abietis extract)	40	NR	NR	NR	NR	NR	NR	NR
Kleeberg, 2004 [70]	Iscador® M (*Viscum*, *mali* extract)	102	8 years	NR	NR	NR	NR	NR/ (5) (Tx discontinuation d/t Grade 3–4 toxicity (WHO classification))	(0) organ toxicity (definition NR)
	No added tx	102	8 years	NR	NR	NR	NR	NR/ (0) (Tx Discontinuation d/t Grade 3–4 toxicity (WHO classification))	(0) organ toxicity (definition NR)
Goebell, 2002 [68]	*V. album* Lectin extract (brand NR)	23	18 mon.	(0) (Adverse events after application of mistletoe lectin) (no specific Definition)	NR	NR	NR	NR	NR
	No added tx	22	18 mon.	NR	NR	NR	NR	NR	NR
Ibrahiem, 2009 [69]	Mistletoe extract (brand NR)	30	NR	(30) Self-limited local skin reaction after first SC dose ^b^ (definition NR) (0) Biochemical or hematological changes (definition NR)	NR	NR	NR	NR	NR
	BCG standard therapy	30	NR	(23) Painful bladder sensation (definition NR)	NR	NR	NR	NR	NR
Steuer- Vogt, 2001 [75]	Eurixor^®^ (mistletoe extract)	97	60 weeks	(47) {At start of tx: pxs with AEs upon injections (local and/or systemic side-effects upon mistletoe injections into abdominal wall. Most common local AEs were rubor and prurigo)} (4) {At 32 weeks or later: pxs with AEs upon injections (local and/or Systemic side-effects upon mistletoe injections into abdominal wall}	NR	NR	NR	(16) Pxs who refused further injections because of mistletoe -induced AEs (definition NR)	(4) Generalized drug reactions (melalgia, fever ≤ 39 °C, sleeplessness, tiredness, coldness or heat sensation and sneezing)
	No added tx	105	60 weeks	NR	NR	NR	NR	NR	NR

^a^ Pneumonia was regarded as related to etoposide (serious adverse drug reaction). ^b^ Intravesical mistletoe was safe and tolerable. Abbreviations: AE = adverse events, mon. = month, N = number, NR = not reported, OC = other complications not already reported on above, pxs = patients, tx = treatment.

## Data Availability

The data presented in this study are available in detail in the online Supplementary Material for this article.

## Supplementary Materials

The following are available online at https://www.mdpi.com/article/10.3390/curroncol30090595/s1, MEDLINE Search Strategy, Table S1: Characteristics of the 3 nonrandomized and observational Studies, Table S2: Interventions/exposures in the 3 nonrandomized and observational studies, Table S3: Summary characteristics of 7 RCTs and 3 non-RCTs, Table S4: Additional results from RCTs, Table S5: Results from nonrandomized and observational studies.
