# Supplementary material for: Mistletoe Extracts during the Oncological Perioperative Period: A Systematic Review and Meta-Analysis of Human Randomized Controlled Trials
Source: Curr Oncol. 2023 Sep 6;30(9):8196–219. doi: 10.3390/curroncol30090595 (PMC10529072; doi:10.3390/curroncol30090595)
Supplement: Supplementary file 1 [file curroncol-30-00595-s001.zip › curroncol-2539458-supplementary.pdf]

SUPPLEMENTARY APPENDIX FOR:

Mistletoe extracts during the oncological perioperative period: A systematic review and meta-analysis of human randomized controlled trials

Cogo E, Elsayed M, Bhardwaj S, Cooley K, Aycho C, Liang V, Papadogianis P, Psihogios A, Seely D

TABLE OF CONTENTS

MEDLINE Search Strategy 2
Table S1. Characteristics of the 3 Nonrandomized & Observational Studies 3
Table S2. Interventions/Exposures in the 3 Nonrandomized & Observational Studies 4
Table S3. Summary Characteristics of 7 RCTs and 3 non-RCTs 5
Tables S4. Additional results from the RCTs 7
Tables S5. Results from Nonrandomized & Observational Studies 19

ABBREVIATIONS:

AE=adverse event, BID=twice daily, CI=confidence interval, NA=not applicable, No.=number, NR=not reported, OC=other complication, post-op=post-operative, pre-op=pre-operative, QD=once daily, RCT=randomized controlled trial, SD=standard deviation, SE=standard error, TID=three times daily, tx=treatment, var=variance.

MEDLINE Search Strategy

(Ovid; 1946-present)

1. fish oils/ or fatty acids, omega-3/ or docosahexaenoic acids/ or eicosapentaenoic acid/
2. arginine/
3. glutamine/
4. RNA/
5. exp Mistletoe/ or Viscum album/
6. vitamin k/ or vitamin k 1/ or vitamin k 2/ or vitamin k 3/
7. probiotics/ or synbiotics/ or exp Lactobacillus/ or exp Bifidobacterium/ or Saccharomyces boulardii/
8. amino acids, branched-chain/ or isoleucine/ or leucine/ or valine/
9. Lycopersicon esculentum/
10. Ubiquinone/
11. ((fish adj oil*) or (omega adj 3*) or docosahexaenoic or eicosapentaenoic or immunonutrition or arginine or glutamine or mistletoe or Viscum album or (vitamin adj k*) or probiotic* or synbiotic* or Lactobacill* or Bifidobacteri* or Saccharomyces boulardii or branched chain amino acid* or leucine or isoleucine or valine or tomato or lycopene or coq10 or (coenzyme adj q10) or ubiquino*).ti,ab,kf.
12. or/1-11
13. (cancer* or carcinoma* or tumor* or tumour* or neoplas* or adenoma* or metastas* or malignan* or oncolog*).mp.
14. exp Neoplasms/
15. exp Precancerous Conditions/
16. 14 not 15
17. ((anti adj cancer) or anticancer). ti,ab,kf.
18. ((anti adj neoplastic) or antineoplastic). ti,ab,kf.
19. exp Antineoplastic Agents/
20. 13 or 16 or 17 or 18 or 19
21. 12 and 20
22. limit 12 to cancer
23. 21 or 22
24. exp perioperative care/ or exp perioperative period/ or exp Specialties, Surgical/ or exp Surgical Procedures, Operative/ or exp Postoperative Complications/
25. (surgery or surgical or surgeries or resect* or perioperative* or preoperative* or operative* or postoperative*). ti,ab,kf.
26. 24 or 25
27. 23 and 26
28. exp Animals/ not (exp Animals/ and Humans/)
29. 27 not 28
30. limit 29 to english language
31. (randomized controlled trial or controlled clinical trial).pt.
32. (randomized or randomly or placebo).ab.
33. (trial or groups).ab.
34. drug therapy.fs.
35. 31 or 32 or 33 or 34
36. 30 and 35
37. exp case-control studies/ or exp cohort studies/
38. longitudinal studies/ or follow-up studies/ or prospective studies/ or retrospective studies/ or Control Groups/ or Matched-Pair Analysis/ or Comparative studies/
39. 37 or 38
40. 30 and 39
41. limit 30 to (clinical trial, phase ii or clinical trial, phase iii or clinical trial, phase iv or comparative study or controlled clinical trial or observational study or randomized controlled trial)
42. 36 or 40 or 41

Table S1. Characteristics of the 3 Nonrandomized & Observational Studies

Study Author, Year (Study period)	Country	Funding	Sample size	Surgical period of Mistletoe exposure	Post-op Mistletoe initiation timing [a]	Cancer types	Cancer/ disease severity	Age (y, var.)	Female (%)	Adjunct tx
Bussing	Germany	NR	101	Preoperative	NA	breast cancer	not in final stages of their disease; life expectancy >12 weeks	NR	100	oral contraceptives
Augustin	Germany & Switzerland	private	686	Postoperative	median 2 months, range 1-4 months	malignant melanoma	UICC/AJCC stages II–III (mostly stage II)	53, NR	56	immunotherapy
Elsasser-Beile	Germany	NR	48	Postoperative	between 2 and 7 weeks after transurethral resection	superficial bladder cancer	pTa G2 or pT1 G2 lesions, which were treated with complete transurethral resection,	NR	23	NR

[a] Denotes how long after surgery the ME was started.
Abbreviations: NA=not applicable, NR=not reported, tx=treatment, var=variance, y=year.

Table S2. Interventions/Exposures in the 3 Nonrandomized & Observational Studies

Study Author, Year	Group/ Brand name	Ingredients & Comparators & Dose per unit	Dosage freq-uency	Route of admin	Tx duration Pre-op	Tx duration Post-op	Tx duration TOTAL
Bussing 2005	Viscum album L. extract. (Heilmittel, Schwäbisch Gmünd, Germany	1 mg mistletoe lectin-standardized infusion along with sodium chloride or Ringer solution, one hour before surgery. 5-mg ampoules provided by Weleda Heilmittel, Schwäbisch Gmünd, Germany. mistletoe lectin (ML) content was 210-290 ng/ml (ML II reference).	Once	IV	single Infusion over 1 hour	NA	1 hour
	Control	NA (no added tx)	NA	NA	NA	NA	NA
Augustin 2005	Iscador	mistletoe extract &NR	3x a week	injection	NA	30 months	30 months
	Control	no added tx	NA	NA	NA	NA	NA
Elsasser-Beile 2005	Mistletoe	Mistletoe	weekly	intravesicle	other	NA	6 weeks
	Bacillus Calmette-Guerin (BCG)	Standard care/ NR	weekly	intravesicle	other	NA	6 weeks

[a] Dosages are presented when they were reported in the studies.

Abbreviations: IV=intravenous, NA=not applicable, Post-op=post-operatively, Pre-op=pre-operatively, tx=treatment

Table S3. Summary Characteristics of 7 RCTs and 3 non-RCTs

		RCTs (7)		Non RCTs -Observational (3)	
Characteristic		N	%	N	%
**Year of publication**					
	2000-2009	6	85	3	100
	2010-2020	1	14	0	0
**Country**					
	Germany	3	43	2	67
	Mixed European countries including Germany[a]	1	14	1	33
	Romania	1	14	0	0
	Italy	1	14	0	0
	Egypt	1	14	0	0
**Cancer types**					
	Bladder	2	29	1	33.3
	Digestive/colon	2	29		
	Osteosarcoma	1	14		
	Melanoma	1	14	1	33.3
	Head and Neck	1	14		
	breast	0	0	1	33.3
**Surgical period of mistletoe use**					
	Pre-operative only	0	0	1	33
	Intra-operative only	1	14	0	0
	Post-operative only	4	57	2	67

	Mixed periods	2	29	0	0
**Mistletoe route of administration**					
	Subcutaneous	5	72	1[b]	33.3
	Intravenous	1	14	1	33.3
	Intravesical	1	14	1	33.3
**Age (mean/median)**					
	33-51 years	1	14	0	33
	52-59 years	3	43	1	0
	60-71 years	2	29	0	0
	Not reported	1	14	2	67
**Female (mean/median)**					
	0-29%	2	29	1	33.3
	30-46%	4	57	1	33.3
	47-100%	0	0	1	33.3
	Not reported	1	14	0	
**Funding**					
	Public	3	43	0	
	Private	1	14	1	33
	Mixed	1	14	0	
	None	1	14	0	
	Not reported	1	14	2	67
**Sample size**					
	19-49	3	43	1	33
	50-99	2	29	0	0
	100-204	2	29	1	33
	More than 204			1	33

[a] Included 13 countries: Germany; France; Switzerland; Austria; Belgium; Great Britain; Yugoslavia; Israel; Czechia; Estonia; Spain; Greece; Poland

[b] reported as injection.

Tables S4 – additional results from the RCTs

Alphabetical of the RCTs

15. Enesel, M.B.; Acalovschi, I.; Grosu, V.; Sbarcea, A.; Rusu, C.; Dobre, A.; Weiss, T.; Zarkovic, N. Periopera- 666 tive application of the Viscum album extract Isorel in digestive tract cancer patients. Anticancer Res 2005, 667 25, 4583-4590.

68. Goebell, P.J.; Otto, T.; Suhr, J.; Rübben, H. Evaluation of an unconventional treatment modality with mistletoe lectin to prevent recurrence of superficial bladder cancer: a randomized phase II trial. J Urol 2002, 168, 72-75.

69. Ibrahiem E., H.I. UP-3.040: Mistletoe Extract as an Adjuvant Therapy after Resection of Superficial Bladder Cancer: Prospective Clinical Randomized Study. Urology 2009, October; 74,

70. Kleeberg, U.R.; Suciu, S.; Bröcker, E.B.; Ruiter, D.J.; Chartier, C.; Liénard, D.; Marsden, J.; Schadendorf, D.; Eggermont, A.M. Final results of the EORTC 18871/DKG 80-1 randomised phase III trial. rIFN-alpha2b versus rIFN-gamma versus ISCADOR M versus observation after surgery in melanoma patients with either high-risk primary (thickness >3 mm) or regional lymph node metastasis. Eur J Cancer 2004, 40, 390-402, doi:10.1016/j.ejca.2003.07.004

71. Longhi A, M.E.K.J.J. A randomized study with adjuvant mistletoe versus oral Etoposide on post relapse disease-free survival in osteosarcoma patients. European Journal of Integrative Medicine 2009, 1, 27-33.

72. Longhi, A.; Reif, M.; Mariani, E.; Ferrari, S. A Randomized Study on Postrelapse Disease-Free Survival with Adjuvant Mistletoe versus Oral Etoposide in Osteosarcoma Patients. Evid Based Complement Al-ternat Med 2014, 2014, 210198, doi:10.1155/2014/210198 . Epub 2014 Mar 31.

73. Longhi, A.; Cesari, M.; Serra, M.; Mariani, E. Long-Term Follow-up of a Randomized Study of Oral Etoposide versus Viscum album Fermentatum Pini as Maintenance Therapy in Osteosarcoma Patients in Complete Surgical Remission after Second Relapse. Sarcoma 2020, 2020, 8260730, doi:10.1155/2020/8260730.eCollection 2020.

74. Schink, M.; Tröger, W.; Dabidian, A.; Goyert, A.; Scheuerecker, H.; Meyer, J.; Fischer, I.U.; Glaser, F. Mis-tletoe extract reduces the surgical suppression of natural killer cell activity in cancer patients. a ran-domized phase III trial. Forsch Komplementmed 2007, 14, 9-17, doi:10.1159/000098135. Epub 2007 Mar 6.

75. Steuer-Vogt, M.K.; Bonkowsky, V.; Ambrosch, P.; Scholz, M.; Neiss, A.; Strutz, J.; Hennig, M.; Lenarz, T.; Arnold, W. The effect of an adjuvant mistletoe treatment programme in resected head and neck cancer patients: a randomised controlled clinical trial. Eur J Cancer 2001, 37, 23-31, doi:10.1016/s0959-8049(00)00360-9

Table for Mortality (RCTs)

Author Year	Tx Name	Sample size	No. deaths	Follow-up time	Survival Outcome	p value
Kleeberg 2004	Iscador® M (*Viscum, mali* extract)	102	60[a]	8 years	all-cause mortality	p>0.05
	No added tx	102	54	8 years	all-cause mortality	
Goebell 2002	*V. album* lectin extract (brand NR)	23	0	18 months	death	p>0.05
	No added tx	22	1	18months	death [b]	
Steuer-Vogt 2001	Eurixor® (mistletoe extract)	97	14	4 years [c]	Tumour-related death	p>0.05
	No added tx	105	20	4 years	Tumour-related death	
Longhi 2020	Iscador® P (*V. album* fermentatum Pini extract)	9	0	12 months	all-cause mortality	p>0.05
	Etoposide	11	3	12 months	all-cause mortality	

[a] HR (adjusted)=1.27 (95% CI: 0.87, 1.84)
[b] One patient died without evidence of tumor or therapy related reasons
[c] Median
Abbreviations: F=follow-up, No=number, NR=not reported, tx=treatment

Table for Recurrence (including distant metastases) (RCTs)

Author Year	Tx Name	Sample size	No. Rec.	Follow-up time	Outcome	p values
Kleeberg 2004	Iscador® M (*Viscum, mali* extract)	102	69	8 years	Recurrence	p=0.10
	No added tx	102	60	8 years	Recurrence	
Goebell 2002	*V. album* lectin extract (brand NR)	23	14	18 months	Recurrence	p>0.05
	No added tx	22	12	18 months	Recurrence	
Ibrahiem 2009	Mistletoe extract (brand NR)	30	5	NR	Invasive bladder cancer development	p>0.05
	BCG standard therapy	30	5	NR	Invasive bladder cancer development	
	Mistletoe extract (brand NR)	30	22	NR	Local recurrence	p=0.003
	BCG Standard therapy	30	9	NR	Local recurrence	
	Mistletoe extract (brand NR)	30	14	NR	Stage and grade progression	p=0.01
	BCG Standard therapy	30	4	NR	Stage and grade progression	
Steuer-Vogt 2001	Eurixor® (mistletoe	97	24 [a]	4 years [b]	All relapses (local/	p>0.05

	extract)				locoregional recurrences, distant metastases and/or secondary tumours)	
	No added tx	105	35	4 years	All relapses (local /locoregional recurrences, distant metastases and/or secondary tumours)	
Longhi 2020	Iscador® P (*V. album* fermentatum Pini extract)	9	4	1 year	Relapse	p>0.05
	Etoposide	11	8	1 year	Relapse	

[a] Disease free survival was HR= 0.627 (95% CI, 0.326; 1.087) for mistletoe vs control (adjustment due to tumour staging, primary tumour site, centre, grading).

[b] Median

Abbreviations: F=follow-up, No=number, NR=not reported, Rec= recurrences tx=treatment

Table for Quality of Life (RCTs)

Study Author, Year	Tx Name	Sample size-F.	Mean value - F. [a]	Outcome Var. type	Var. value-F.	Scale name	Scale range	F-up time	Name of outcome
Longhi 2014	Iscador® P (*V. album* fermentatum Pini extract)	9	11.17	95% CI	2.64; 19.72	EORTC QLQ-C30 scale	NR	12 mos	Global health scale (overall QoL) as change from baseline
	Etoposide	10	3. 51	95% CI	-3.51; 10.54	EORTC QLQ-C30 scale	NR	12 mos	Global health scale (overall QoL) as change from baseline
	Iscador® P (*V. album* fermentatum Pini extract)	9	-0.92	95% CI	-6.42; 4.65	EORTC QLQ-C30 scale	NR	12 mos	Cognitive functioning subscale as change from baseline
	Etoposide	10	-5.94	95% CI	-12.19; 0.31	EORTC QLQ-C30 scale	NR	12 mos	Cognitive functioning subscale as change from baseline
	Iscador® P (*V. album* fermentatum Pini extract)	9	-5.98	95% CI	-10.58; -1.37	EORTC QLQ-C30 scale	NR	12 mos	Emotional functioning subscale as change from baseline
	Etoposide	10	-2.48	95% CI	-9.84; 4.87	EORTC QLQ-C30 scale	NR	12 mos	Emotional functioning subscale as change from baseline
	Iscador® P (*V. album* fermentatum Pini extract)	9	-9. 85	95% CI	-16.31; -3.38	EORTC QLQ-C30 scale	NR	12 mos	Fatigue subscale as change from baseline
	Etoposide	10	1. 13	95% CI	-5.72; 7.99	EORTC QLQ-C30 scale	NR	12 mos	Fatigue subscale as change from baseline
	Iscador® P (*V. album* fermentatum Pini extract)	9	-10.71	95% CI	-18.83; -2.60	EORTC QLQ-C30 scale	NR	12 mos	Pain subscale as change from baseline

	Etoposide	10	10.54	95% CI	4.64; 16.45	EORTC QLQ-C30 scale	NR	12 mos	Pain subscale as change from baseline
	Iscador® P (*V. album* fermentatum Pini extract)	9	7. 3	95% CI	0.15; 14.44	EORTC QLQ-C30 scale	NR	12 mos	Physical functioning subscale as change from baseline
	Etoposide	10	-2.45	95% CI	-8.93; 4.03	EORTC QLQ-C30 scale	NR	12 mos	Physical functioning subscale as change from baseline
	Iscador® P (*V. album* fermentatum Pini extract)	9	3.	95% CI	-7.94; 15.54	EORTC QLQ-C30 scale	NR	12 mos	Role functioning subscale as change from baseline
	Etoposide	10	-6. 31	95% CI	-18.28; 5.65	EORTC QLQ-C30 scale	NR	12 mos	Role functioning subscale as change from baseline
	Iscador® P (*V. album* fermentatum Pini extract)	9	11. 76	95% CI	4.64; 18.88	EORTC QLQ-C30 scale	NR	12 mos	Social functioning subscale as change from baseline
	Etoposide	10	4.78	95% CI	0.51; 9.05	EORTC QLQ-C30 scale	NR	12 mos	Social functioning subscale as change from baseline
	Iscador® P (*V. album* fermentatum Pini extract)	9	**0. 43**	95% CI	-2.7; 3.56	EORTC QLQ-C30 scale	NR	12 mos	Nausea/vomiting subscale as change from baseline
	Etoposide	10	5. 47	95% CI	0.28;10.66	EORTC QLQ-C30 scale	NR	12 mos	Nausea/vomiting subscale as change from baseline
	Iscador® P (*V. album* fermentatum Pini extract)	9	12.63	95% CI	-16.94;8.32	EORTC QLQ-C30 scale	NR	12 mos	Dyspnoea subscale as change from baseline
	Etoposide	10	5.82	95% CI	-1.04;12.68	EORTC QLQ-C30 scale	NR	12 mos	Dyspnoea subscale as change from baseline
	Iscador® P (*V. album* fermentatum Pini extract)	9	11.35	95% CI	-20.74;-1.96	EORTC QLQ-C30 scale	NR	12 mos	Insomnia subscale as change from baseline
	Etoposide	10	5.79	95% CI	2.95;14.53	EORTC QLQ-C30	NR	12 mos	Insomnia subscale

						scale			as change from baseline
	Iscador® P (*V. album* fermentatum Pini extract)	9	-6.4	95% CI	- 6.40;-6.40	EORTC QLQ-C30 scale	NR [b]	12 mos	Appetite loss subscale as change from baseline
	Etoposide	10	1.41	95% CI	-2.15;4.96	EORTC QLQ-C30 scale	NR	12 mos	Appetite loss subscale as change from baseline
	Iscador® P (*V. album* fermentatum Pini extract)	9	-5.54	95% CI	-13.58;2.50	EORT C QLQ-C30 scale	NR	12 mos	Constipation subscale as change from baseline
	Etoposide	10	-0.62	95% CI	-9.65;8.41	EORTC QLQ-C30 scale	NR	12 mos	Constipation subscale as change from baseline
	Iscador® P (*V. album* fermentatum Pini extract)	9	0.83	95% CI	-2.81;4.47	EORTC QLQ-C30 scale	NR	12 mos	Diarrhea subscale as change from baseline
	Etoposide	10	2.44	95% CI	-1.92;6.80	EORTC QLQ-C30 scale	NR	12 mos	Diarrhea subscale as change from baseline
	Iscador® P (*V. album* fermentatum Pini extract)	9	-11.46	95% CI	-16.21;-6.70	EORTC QLQ-C30 scale	NR	12 mos	Financial problems subscale as change from baseline
	Etoposide	10	-2.53	95% CI	-6.88;1.83	EORTC QLQ-C30 scale	NR	12 mos	Financial problems subscale as change from baseline
Enesel 2005	Isorel® A (V. album grown on abietis extract)	40	22.45	NR	NR	Karnofsky Performance Index	NR	60 days	Overall health status as % change from baseline
	No added tx	30	-12.81	NR	NR	Karnofsky Performance Index	NR	60 days	Overall health Status as % change from baseline

[a] Estimated changes: Estimates resulting from a linear fixed model, including base line score, treatment and visit as fixed factors, and patients as random factors.
[b] All postbaseline values in the Viscum group were 0, therefore no test statistic could be calculated

Abbreviations: F=follow-up, NR=not reported, tx=treatment, var=variance, mos =months.

Table for Immune Cells and Inflammatory Markers (RCTs)

Author Year	Tx Name	Sample size-	Mean Value ± SD - Baseline.	Mean Value ±SD Follow up	Unit of outcome	Follow up time	Name of outcome
Schink 2007	Iscador® M special (mistletoe extract)	11	35420± 8909	21772± 8294	Count/cell	7days	Monocyte expression of HLA-DR antigen[a]
	No added tx	11	35405±10470	23744±7351	Count/cell	7days	Monocyte expression of HLA-DR antigen
	Iscador® M special (mistletoe extract)	11	15.2 ±4.9	17.6 ±7.6	%	7 days	NK cell activity [b]
	No added tx	11	15.3 ±2.1	12.3 ±3.9	%	7 days	NK cell activity
Enesel 2005[c]	No added tx	30	NR	0.43NR	% change	14days	B Lymphocytes[d]
	Isorel® A (*V. album* grown on *abietis* extract)	40	NR	9.73<br>NR	% change	14 days	B Lymphocytes
	No added tx	30	NR	-0.16<br>NR	% change	14 days	T Lymphocytes (using Rosette E method) [d]
	Isorel® A (*V. album* grown on *abietis* extract)	40	NR	6.89<br>NR	% change	14 days	T Lymphocytes (using Rosette E method)
	No added tx	30	NR	-1.1<br>NR	% change	14 days	CD3 (using flow cytometry) [d]
	Isorel® A (*V. album* grown on *abietis* extract)	40	NR<br>NR<br>NR	10.54<br>NR	% change	14 days	CD3 (using flow cytometry)
	No added tx	30	NR	-4.4<br>NR	% change	14 days	CD4[d]

	Isorel® A (*V. album* grown on *abietis* extract)	40	NR	18.46<br>NR	% change	14 days	CD4
	No added tx	30	NR	-6.84<br>NR	% change	14 days	CD4/CD8 ratio[d]
	Isorel® A (*V. album* grown on *abietis* extract)	40	NR	25.69<br>NR	% change	14 days	CD4/CD8 ratio
	No added tx	30	NR<br>NR<br>NR	3.4<br>NR	% change	14 days	CD8 [d]
	Isorel® A (*V. album* grown on *abietis* extract)	40	NR	-7.28<br>NR	% change	14 days	CD8
	No added tx	30	NR	-1.98<br>NR	% change	14 days	complement C3[d]
	Isorel® A (*V. album* grown on *abietis* extract)	40	NR	20.11<br>NR	% change	14 days	complement C3
	No added tx	30	NR	1.56<br>NR<br>NR	% change	14 days	complement C4[d]
	Isorel® A (*V. album* grown on *abietis* extract)	40	NR	30.01<br>NR	% change	14 days	complement C4
	No added tx	30	NR	-2.8<br>NR	% change	14 days	IgA[d]
	Isorel® A (*V. album* grown on *abietis* extract)	40	NR	27<br>NR	% change	14 days	IgA
	No added tx	30	NR	-2.21<br>NR	% change	14 days	IgG[d]
	Isorel® A (*V. album* grown on *abietis* extract)	40	NR	13.15<br>NR	% change	14 days	IgG
	No added tx	30	NR	-1.8<br>NR	% change	14 days	IgM [d]
	Isorel® A	40	NR	23.31	% change	14 days	IgM

	(*V. album* grown on *abietis* extract)			NR			
	No added tx	30	NR	-19.98<br>NR	% change	14 days	Lymphocytes [d]
	Isorel® A (*V. album* grown on *abietis* extract)	40	NR	18.28<br>NR	% change	14 days	lymphocytes
	No added tx	30	NR	-4.98<br>NR	% change	14 days	NK cells count [d]
	Isorel® A (*V. album* grown on *abietis* extract)	40	NR	44.26<br>NR	% change	14 days	NK cells counts
	No added tx	30	NR	32.72<br>NR	% change	14 days	total leukocytes [d]
	Isorel® A (*V. album* grown on *abietis* extract)	40	NR	16.9<br>NR	% change	14 days	total leukocytes
Longhi 2020[c]	Iscador® P (*V. album* fermentatum Pini extract)	7	NR	–18.63±40.76	mm^3	12mons	Change of CD19 count from baseline [e]
	Etoposide	7	NR	72.34 ±SD 87.55	mm^3	12 mons	change of CD19 count from baseline
	Iscador® P (*V. album* fermentatum Pini extract)	7	NR	–39.82 ±102.51	mm^3	12 mons	Change of CD4 count from baseline [e]
	Etoposide	7	NR	26.62 ±121.35	mm^3	12 mons	change of CD4 count from baseline
	Iscador® P *V. album* fermentatum Pini extract)	7	NR	–24.59 ±180.06	mm^3	12 mons	change of CD56 count from baseline [e]
	Etoposide	7	NR	17.45 ±84.27	mm^3	12 mons	change of CD56 count from baseline
	Iscador® P *V. album* fermentatum Pini extract)	7	NR	–20.38 ±101.57	mm^3	12 mons	change of CD8 count from baseline [e]

	Etoposide	7	NR	–32.52 ±102.56	mm^3	12 mons	change of CD8 count from baseline
	Iscador® P (V. *album* fermentatum Pini extract)	7	NR	–85.60 ±409.01	mm^3	12 mons	change of total lymphocyte count from baseline [e]
	Etoposide	7	NR	119.25 ±248.97	mm^3	12 mons	change of total lymphocyte count from baseline
	Iscador® P (V. *album* fermentatum Pini extract)	7	NR	–35.13 ±209.23	mm^3	12 mons	change of CD3 count from baseline [e]
	Etoposide	7	NR	61.09 ±167.88	mm^3	12 mons	change of CD3 count from baseline

[a] Definition from the study: Measured using flow cytometric antibodies bound per cell method. There was no significant difference between the groups p=0.46

[b] Definition from the study: Cytotoxic killing against K 562 tumour cells using LDH assay (Korzeniewsk 1983). The Nk cell activity was significantly lower in mistletoe group than in the control group p=0.04 for the post to pre differences.

[c] Non of the biomarkers were statistically evaluated for a difference between the groups.

[d] Definition from the study: % change from baseline.

[e] Definition was nor reported

Abbreviations: B=baseline, F=follow-up, NR=not reported, tx=treatment, var=variance, mos =months

Table for - Blood loss (RCTs)

Study Author, Year	Treatment Name	Sample size	Number of events	Name of outcome
Schink 2007	Iscador® M special (mistletoe extract)	12	1	Perioperative blood transfusion
	No added treatment	14	3	Perioperative blood transfusion

Tables S5. Results from Nonrandomized & Observational Studies

Alphabetical List of the Included Nonrandomized & Observational Studies

NB: The number below corresponds to the reference number cited in the main report's References section:

76. Elsässer-Beile, U.; Leiber, C.; Wetterauer, U.; Bühler, P.; Wolf, P.; Lucht, M.; Mengs, U. Adjuvant in-travesical treatment with a standardized mistletoe extract to prevent recurrence of superficial urinary bladder cancer. Anticancer Res 2005, 25, 4733-4736.

77. Augustin, M.; Bock, P.R.; Hanisch, J.; Karasmann, M.; Schneider, B. Safety and efficacy of the long-term adjuvant treatment of primary intermediate- to high-risk malignant melanoma (UICC/AJCC stage II and III) with a standardized fermented European mistletoe (Viscum album L.) extract. Results from a multicenter, comparative, epidemiological cohort study in Germany and Switzerland. Arzneimittel-forschung 2005, 55, 38-49, doi:10.1055/s-0031-1296823

78. Büssing, A.; Bischof, M.; Hatzmann, W.; Bartsch, F.; Soto-Vera, D.; Fronk, E.M.; Gmeindl, M.; Stein, G.M. Prevention of surgery-induced suppression of granulocyte function by intravenous application of a fermented extract from Viscum album L. in breast cancer patients. Anticancer Res 2005, 25, 4753-4757.

Table of Nonrandomized or Cohort Studies for Mortality

Author Year	Tx Name	Tx sample size	No. deaths [a]	Follow-up time
Augustin 2005	mistletoe extract	329	29	81 months
	no added tx	357	38	52 months

[a] Adjusted HR for Overall Survival= 0.64 (95% CT, 0.42 to 0.96)

Abbreviations: No= number, tx=treatment,

Table of Nonrandomized or observational Studies for Recurrence

Author Year	Tx Name	Tx sample size	No. recurrences	Follow-up time Follow-up unit
Elsasser-Beile 2005	An aqueous mistletoe extract standardized to the content of mistletoe lectin	30	9	12 months
	Bacillus Calmette-Guerin (BCG)	18	5	12 months

Abbreviations: No=number, tx=treatment, var=variance, mos =months

Table of Nonrandomized or observational studies for Metastases

Author Year	Tx Name	Tx sample size	No. metastasis	Follow-up time [a]	Name of outcome
Augustin 2005	mistletoe extract	329	73	81 months	overall metastases
	no added tx	357	89	52 months	overall metastases
	mistletoe extract	329	10	81 months	brain metastases
	no added tx	357	15	52 months	brain metastases
	mistletoe extract	329	18	81 months	lung/mediastinal metastases
	no added tx	357	37	52 months	lung/mediastinal metastases
	mistletoe extract	329	39	81 months	lymph node metastases
	no added tx	357	62	52 months	lymph node metastases

[a] each arm reported a different median follow-up time.
Abbreviations: No=number, tx=treatment,

Table of Nonrandomized or observational studies for Immune and inflammatory markers

Author year	Tx Name	Tx sample size-Baseline	Mean value-Baseline	Outcome variance type	Variance value-Baseline	Tx sample size-Follow up	Mean value-Follow up	Outcome var. type	Variance value-Follow up	Unit of outcome	Follow-up time	Name of outcome
Bussing 2005	mistletoe extract	50	0.91	SD	0.39	50	1.71	SD	1.5	mcg/l	3 days	C-reactive protein
	no added tx	51	1.01	SD	0.96	51	2.96	SD	3.26	mcg/l	3 days	C-reactive protein
	mistletoe extract	50	6.87	SD	1.98	50	6.9	SD	1.97	x1,000/microL	3 days	leukocyte numbers
	no added tx	51	6.88	SD	1.63	51	7.02	SD	1.88	x1,000/microL	3 days	leukocyte numbers
	mistletoe extract	50	1.95	SD	0.53	50	1.87	SD	0.64	x1,000/microL	3 days	lymphocyte numbers
	no added tx	51	1.97	SD	0.64	51	1.85	SD	0.62	x1,000/microL	3 days	lymphocyte numbers

Abbreviations: No=number, tx=treatment, var=variance

Table for Adverse Events and Other Complications in Non-RCTs

Study Author, Year	Tx Name	Sample size	Follow-up time & unit	All adverse effects (AEs)/most common AEs [N] (definition)	Serious AEs [N] (definition)	Severe AEs [N] (definition)	Surgical Complication [N] (definition)	Drug reduction /Treatment Discontinuation Number (definition)	OC [N] (definition) –
Bussing 2005,	mistletoe extract	50	3 days	[0] mistletoe-related side-effects (definition NR)	NR	NR	NR	NR	NR
	no added tx	51	NR	[NR] (definition NR)	NR	NR	NR	NR	NR
Augustin, 2005	mistletoe extract	329	81 months	[11][a] systemic adverse drug reactions (ADRs) attributed to the mistletoe treatment. Definition [a]	[0] life-threatening ADRs (definition NR)	NR	NR	[42][b] local reaction at the injection site definition [b] [6] Mistletoe termination due to ADRs definition NR	NR
	no added tx	357	NR	NR	NR	NR	NR	NR	NR

Elsasser-Beile , 2005	aqueous mistlet ract standardized content of mistle lectin	30	2 months	[0] local or systemic side-effects definition [c]	NR	NR	NR	NR	NR
	us Calmette-Gue (BCG)	18	NR	NR	NR	NR	NR	NR	NR

[a][headache (4), fatigue (3), fever (3), allergy (1), itching (1), Quincke's edema (1), exanthema (1), eczema (1),"sick feeling" (1), loss of hair (1), sleep disturbance (1), dyspnea (1), and pancreatitis recurrence (1)]

[b] [erythema (41), edema (12), itching or local pain (3), or other local reactions (3)]

[c] Toxicity WHO grades 1-4
